# Review of machine learning methods for RNA secondary structure prediction

**DOI:** 10.1371/journal.pcbi.1009291

**Published:** 2021-08-26

**Authors:** Qi Zhao, Zheng Zhao, Xiaoya Fan, Zhengwei Yuan, Qian Mao, Yudong Yao

**Affiliations:** 1 College of Medicine and Biological Information Engineering, Northeastern University, Shenyang, Liaoning, China; 2 School of Information Science and Technology, Dalian Maritime University, Dalian, Liaoning, China; 3 School of Software, Key Laboratory for Ubiquitous Network and Service Software of Liaoning Province, Dalian University of Technology, Dalian, Liaoning, China; 4 Key Laboratory of Health Ministry for Congenital Malformation, Shengjing Hospital of China Medical University, Shenyang, Liaoning, China; 5 College of Light Industry, Liaoning University, Shenyang, Liaoning, China; 6 Key Laboratory of Agroproducts Processing Technology, Changchun University, Changchun, Jilin, China; 7 Department of Electrical and Computer Engineering, Stevens Institute of Technology, Hoboken, New Jersey, United States of America; University of Missouri, UNITED STATES

## Abstract

Secondary structure plays an important role in determining the function of noncoding RNAs. Hence, identifying RNA secondary structures is of great value to research. Computational prediction is a mainstream approach for predicting RNA secondary structure. Unfortunately, even though new methods have been proposed over the past 40 years, the performance of computational prediction methods has stagnated in the last decade. Recently, with the increasing availability of RNA structure data, new methods based on machine learning (ML) technologies, especially deep learning, have alleviated the issue. In this review, we provide a comprehensive overview of RNA secondary structure prediction methods based on ML technologies and a tabularized summary of the most important methods in this field. The current pending challenges in the field of RNA secondary structure prediction and future trends are also discussed.

## Introduction

Since its discovery, for a long time, RNA was regarded solely as a message carrier between DNA and protein. However, we are now beginning to understand its important roles, as increasing numbers of noncoding RNAs (ncRNA) are being discovered [1]. According to the latest report, less than 2% of the human genome comprises protein-coding regions [2]. The majority of the remaining genome portions encode ncRNAs [3], which are involved in translation, catalysis, RNA stability, RNA modification, gene expression regulation, protein synthesis, and protein degradation [4–9]. The enormous importance of ncRNAs in various human diseases, such as cancer, diabetes, and atherosclerosis [6,10], is also being recognized.

ncRNA molecules often fold into higher-order structures, and functionally important ncRNA structures are typically conserved during evolution. Similar to protein, the ncRNA function is usually closely related to its structure. Currently, a wide variety of ncRNA sequences are publicly available, and their numbers keep dramatically increasing [11]. By contrast, most of their structures remain unknown, which hinders the inference of their function. Hence, efficient determination of ncRNA structure is of great value to research.

**Fig 1:**
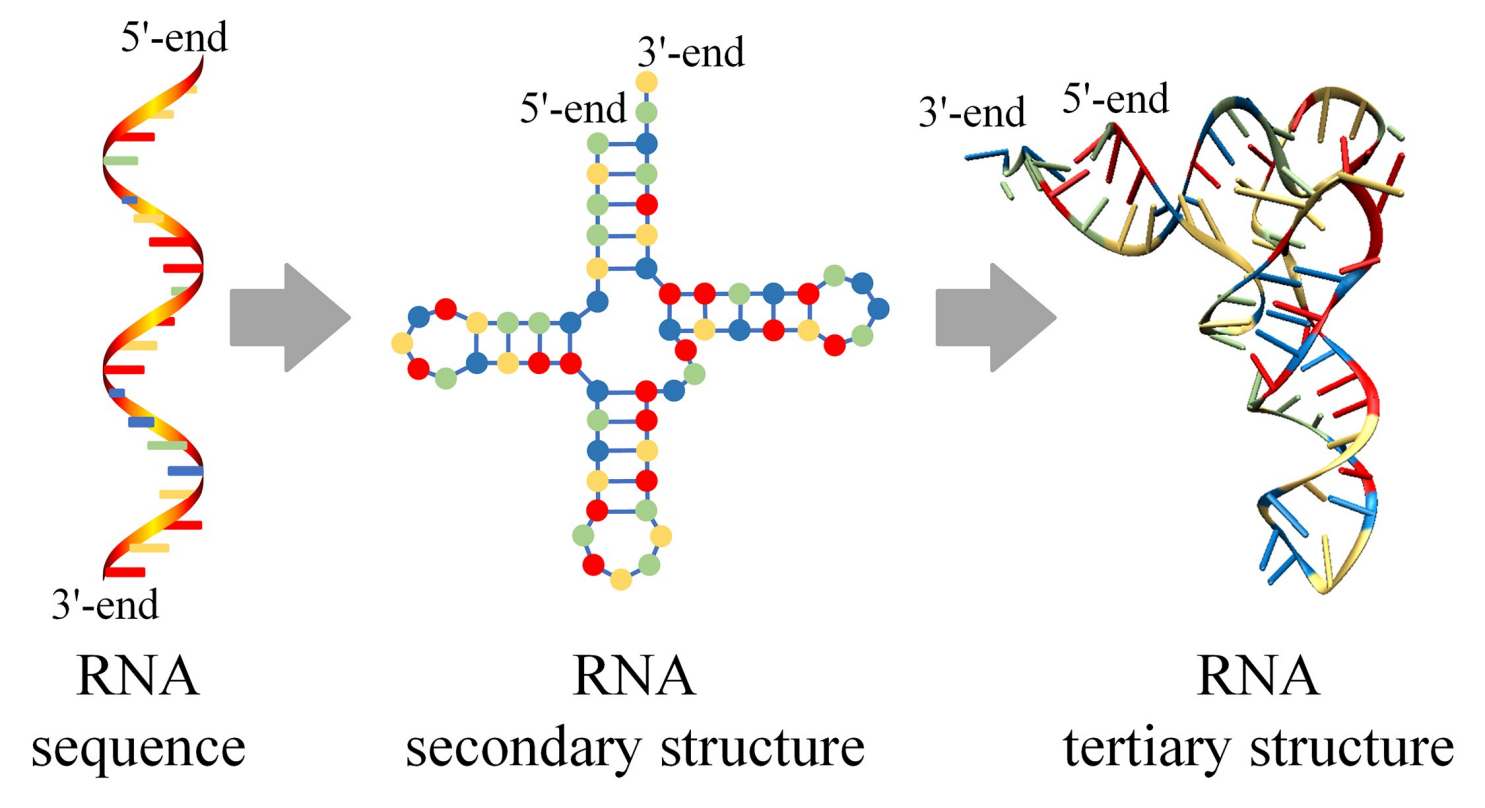
RNA primary (left), secondary (middle), and tertiary structures (right). The RNA folding process is hierarchical, i.e., the RNA secondary structure forms rapidly from linear RNA (primary structure) with a large energy loss, while the formation of a complex tertiary structure is usually much slower.

Unlike the global folding of protein driven by hydrophobic forces, the RNA folding process is hierarchical [12] (Fig 1). Specifically, the RNA secondary structure, composed of base pairs, forms rapidly from linear RNA (primary structure), with a large energy loss, while the formation of a complex tertiary structure (or 3D structure) is usually much slower [13]. The RNA secondary structure is very stable and abundant in the cell, which is important for ncRNA function [14,15]. Even without the knowledge of the higher-order structure, RNA secondary structure is sufficient to infer function and for other practical applications [15].

Computational predictions are mainstream approaches for identifying RNA secondary structure. A number of prediction methods have been developed since the 1970s. Most of these methods attempt to identify a structure with a minimum free energy (MFE), in agreement with the hypothesis that an RNA molecule is likely to exist in an MFE state, just like protein [16]. Many prominent software applications have been developed incorporating these methods [17–19]. However, in the last 10 years, the accuracy of prediction failed to significantly improve, and neither did the calculating speed. An alternative approach, the machine learning (ML)-based methodology, was proposed to improve the predictions of RNA secondary structure. However, such methods did not receive much attention because of the limited accuracy. That was mainly because of the small size of the training datasets and the limitations of simple ML models. As a result of the recent explosion of RNA sequence data and the improvement of ML techniques, especially deep learning (DL) techniques, the latest ML-based methods supersede the current mainstream methods in terms of accuracy and applicability. We believe that these ML-based methods will inspire the next generation of prediction tools in the near future.

In this paper, we provide a comprehensive overview of ML-based methods for RNA secondary structure prediction, with a thorough discussion of their advantages and disadvantages. We also provide a tabularized summary (Table 1) of the most important models in the field, and a perspective on the future promising directions, with a special emphasis on DL models. Although several review papers have been published on the topic of RNA secondary structure prediction [20–22], reviews with an emphasis on ML techniques are lacking. We believe that this review will enable researchers to understand the key issues that remain to be solved and facilitate further advances in predicting the RNA secondary structures based on ML.

**Table 1:** Summary of the ML-based RNA secondary structure prediction methods.

Category		Title	Date	Author	ML Technique	Resource	Reference
Score scheme based on ML model	Free energy parameter-refining approach based on ML	Thermodynamic Parameters for an Expanded Nearest-Neighbor Model for Formation of RNA Duplexes with Watson-Crick Base Pairs	1998	Xia et al.	Linear regression	Table 1 in the paper (https://pubs.acs.org/doi/10.1021/bi9809425#)	[56]
		Efficient parameter estimation for RNA secondary structure prediction	2007	Andronescu et al.	Constraint generation	http://www.rnasoft.ca/CG/	[73]
		Computational approaches for RNA energy parameter estimation	2010	Andronescu et al.	Loss-augmented max-margin constraint generation model, Boltzmann-likelihood model	http://www.cs.ubc.ca/labs/beta/Projects/RNA-Params	[74]
	Weighted approach based on ML	Rich Parameterization Improves RNA Structure Prediction	2011	Zakov et al.	Discriminative structured-prediction learning framework combined, online learning algorithm	http://www.cs.bgu.ac.il/?negevcb/contextfold	[77]
		A Max-Margin Training of RNA Secondary Structure Prediction Integrated with the Thermodynamic Model	2018	Akiyama et al.	SSVM	https://github.com/keio-bioinformatics/mxfold	[78]
		RNA secondary structure prediction using deep learning with thermodynamic integration	2021	Sato et al.	Deep neural network	http://www.dna.bio.keio.ac.jp/mxfold2/	[79]
	Probabilistic approach based on ML	Stochastic context-free grammars for tRNA modeling	1994	Sakakibara et al.	EM method	-	[29]
		RNA secondary structure prediction using stochastic context-free grammars and evolutionary history	1999	Knudsen and Hein	EM method	-	[82]
		Pfold: RNA secondary structure prediction using stochastic context-free grammars	2003	Knudsen and Hein	EM method		[81]
		CONTRAfold: RNA secondary structure prediction without physics-based models	2006	Do et al.	CLLM	http://contra.stanford.edu/contrafold/	[86]
		A semi-supervised learning approach for RNA secondary structure prediction	2015	Yonemoto et al.	Semi-supervised learning algorithm	-	[87]
Preprocessing and postprocessing based on ML model	Preprocessing based on ML model	A tool preference choice method for RNA secondary structure prediction by SVM with statistical tests	2013	Hor et al.	SVM	-	[88]
		Research on folding diversity in statistical learning methods for RNA secondary structure prediction	2018	Zhu et al.	Statistical context-free grammar model	-	[89]
	Postprocessing based on ML model	Using a neural network to identify secondary RNA structures quantified by graphical invariants	2008	Haynes et al.	MLP	-	[90]
		A predictive model for secondary RNA structure using graph theory and a neural network	2010	Koessler et al.	MLP	-	[91]
Predicting process based on ML model	End-to-end approach	Parallel algorithms for finding a near-maximum independent set of a circle graph	1990	Takefuji et al.	System composed of several interactional neurons	-	[92]
		An Hopfield Neural Network-Based Algorithm for RNA Secondary Structure Prediction	2006	Liu et al.	Hopfield networks	-	[93]
		Secondary Structure Prediction of RNA using Machine Learning Method	2011	Qasim et al.	MLP	-	[96]
		Neural Networks, Adaptive Optimization, and RNA Secondary Structure Prediction	1993	Steeg	MFT network	-	[94]
		RNA secondary structure prediction by MFT neural networks	2003	Apolloni et al.	MFT network with mean field approximation to update network’s nodes	-	[139]
		RNA secondary structure prediction using an ensemble of two-dimensional deep neural networks and transfer learning	2019	Singh et al.	Compound deep neural networks, transfer learning	https://sparks-lab.org/server/spot-rna/	[97]
		RNA secondary structure prediction by learning unrolled algorithms	2020	Chen et al.	Compound deep neural networks	https://github.com/ml4bio/e2efold	[99]
		Machine learning a model for RNA structure prediction	2020	Calonaci et al.	CNN, MLP	-	[100]
	Hybrid approach	RNA secondary structure prediction from sequence alignments using a network of k-nearest neighbor classifiers	2006	Bindewald et al.	Hierarchical network of k-nearest neighbor model	-	[49]
		Developing parallel ant colonies filtered by deep learned constrains for predicting RNA secondary structure with pseudo-knots	2020	Quan et al.	Bi-LSTM	-	[103]
		RNA Secondary Structure Prediction Based on Long Short-Term Memory Model	2018	Wu et al.	Bi-LSTM	-	[102]
		Predicting RNA secondary structure via adaptive deep recurrent neural networks with energy-based filter	2019	Lu et al.	Bi-LSTM	-	[101]
		A New Method of RNA Secondary Structure Prediction Based on Convolutional Neural Network and Dynamic Programming	2019	Zhang et al.	CNN	-	[104]
		DMfold: A Novel Method to Predict RNA Secondary Structure with Pseudoknots Based on Deep Learning and Improved Base Pair Maximization Principle	2019	Wang et al.	Bi-LSTM	https://github.com/linyuwangPHD/RNA-Secondary-Structure-Database.	[105]
		Improving RNA secondary structure prediction via state inference with deep recurrent neural networks	2020	Willmott et al.	Bi-LSTM	https://github.com/dwillmott/rna-state-inf	[107]

“-”indicates “not available.”

CLLM, conditional log-linear model; CNN, convolutional neural network; EM, expectation-maximization; MFT, mean field theory; ML, machine learning; MLP, multilayer perceptron; SSVM, structured support vector machine; SVM, support vector machine.

## RNA secondary structure: The basics

The RNA molecule is an ordered sequence of nucleotides that contain 1 of the 4 bases: adenine (A), cytosine (C), guanine (G), and uracil (U), arranged in the 5′ to 3′ direction. Pairing (via hydrogen bonds) of these 4 bases within an RNA molecule gives rise to the secondary structure. Typically, each base pairs with at most one other base. The canonical base pairs include the Watson–Crick base pairs (A–U and G–C) and the wobble base pair (G–U). These base pairs often result in the formation of a nested structure, in which multiple stacked base pairs form a helix, and one or multiple unpaired base pairs form a loop.

It has to be noted that 3 kinds of special base pairs [23] commonly occur in the native RNA secondary structures, i.e., noncanonical base pairs, base triples, and pseudoknots. Noncanonical base pairs are the base pairs other than A–U, G–C, and G–U, and they make up 40% of all base pairs in structured RNAs [24]. Base triples are the cluster of 3 bases interacting, which [25] can stabilize many RNA tertiary interactions [26]. Base triples also occur widely in RNA structures. A pseudoknot [27] occurs when bases in different loops pair with each other, forming a nonnested structure between 2 bases that are located apart from each other. Pseudoknots represent a small fraction of base pairs in known RNA secondary structures but often play an important role in RNA function [28].

Typically, the secondary structure of an RNA molecule with a length *n* can be regarded as:

1. A set of base pairs {(*i,j*),1≤*i*<*j*≤*n*}, where (*i,j*) indicates a base pair formed between the *i*-th and *j*-th nucleotide in the RNA sequence; or a set of compatible helixes [28].
2. A contact table (CT table), i.e., a square matrix, with elements in the *i*-th row and *j*-th column representing the interaction between the *i*-th and *j*-th nucleotides in the RNA sequence.
3. A graph, where nodes represent nucleotides and edges represent base pairing relationships.
4. A labeled sequence with the length *n*, e.g., “dot-parenthesis” notation, with matching parentheses for paired bases and dots for unpaired bases.
5. A parse tree derived from context-free grammars, of which the leaf nodes comprise the RNA sequence [29].

The above definitions form the basis of both traditional and ML-based RNA secondary structure prediction methods.

## Traditional methods of detecting or predicting RNA secondary structure

RNA structure determination is a fast-evolving topic. Many different methods have emerged in the last 20 years. They can be divided into 2 categories, i.e., wet lab experimental approaches and computational predicting approaches.

### Wet lab experiments

X-ray crystallography [30] and nuclear magnetic resonance (NMR) [31] are the most accurate approaches for determining RNA structures, both of which can offer structural information at a single base pair resolution. However, both methods are characterized by high experimental cost and low throughput, limiting their wide usage. In addition, RNA molecules are highly unstable and difficult to crystallize. Although many methods have been developed to infer the state of nucleotides (paired or unpaired) in an RNA molecule using enzymatic [32,33] or chemical probes [34,35] coupled with next-generation sequencing [36,37], most of them can only be used to capture the RNA secondary structure in vitro. The obtained structure may differ markedly from the in vivo conformation. In fact, to date, the structure of only a very small percentage (<0.001%) of known ncRNAs has been determined experimentally [38]. Hence, predicting the RNA secondary structure using computational methods is an important alternative to wet lab–based approaches.

### Traditional computational methods

Comparative sequence analysis [39,40] is the most accurate computational method for determining the RNA secondary structure. This method is based on the assumption that the RNA secondary structure is evolutionarily conserved to a greater extent than the RNA sequence. This method usually finds the base pairs that covary to maintain Watson–Crick and wobble base pairs (compensatory mutations) [41] of a given sequence using a set of homologous sequences. Han and Kim [42] designed the first comparative sequence analysis algorithm based on the phylogenetic comparative analysis. This algorithm predicts a common secondary structure conserved in the given homologous sequence set with a high time complexity (*O*(*n*^3^), *n* being the RNA sequence length). To reduce the running time, Tahi and colleagues [43] implemented another algorithm DCfold with time complexity *O*(*n*^2^ log *n*). DCfold searches for helices based on their lengths and mutation rates using a “divide and conquer” approach. Comparative sequence analysis can also predict the structures with pseudoknots [44–46]; however, the accuracy is very limited. In addition, comparative sequence analysis can be combined with score-based methods [47–50], e.g., RNAalifold [48], KnetFold [49], and ILM [47]. One great limitation of this method is that it requires a large set of homologous sequences. However, only thousands of RNA families are currently known [51], which makes it impossible to obtain homologous sequences for all RNAs. Therefore, most methods for RNA secondary structure prediction are score based, where only a single RNA sequence is required as the input.

Score-based methods are the most widely used methods and have dominated the field of RNA secondary structure prediction in the last 4 decades. These methods assume that the native RNA structure is a structure with a minimum/maximum total score, depending on the hypothesis of RNA folding mechanism or its simplification. Hence, the problem of RNA secondary structure prediction is transformed into an optimization problem. Since the RNA secondary structure can be recursively broken down into smaller elements with independent score contributions, the dynamic programming (DP) algorithm is often employed to identify the optimal structure. Evaluation of the score for structure elements requires a score scheme of many parameters. Nussinov and Jacobson [52] proposed the first, and also the simplest, DP algorithm for finding the maximum-matching structure. The authors proposed to assign one point to each matched base pair and assumed that the native structure is the structure with the maximum score among all the possible conformations. Zuker and Stiegler [53] proposed a more realistic scoring scheme based on free energy, the nearest neighbor model (NN model) [54–57]. It is based on Tinoco’s hypothesis (see Section 4.1) [58]. The NN model can be used for the calculation of energy changes of any structure of a given RNA molecule, and the DP algorithm can be also employed to efficiently find the MFE structure. A number of slightly different variations of this method were also proposed [59–62]. For predicting the structure with noncanonical base pairs, some other score schemes were employed as scoring functions, such as nucleotide cyclic motifs score system [63–65] or equilibrium partition function [66]. In addition, several score-based methods were developed to predict RNA secondary structures with pseudoknots [67–71], where the structure search scope or input RNA length is limited or the types of pseudoknots are restricted to lower the time complexity in general.

However, the folding mechanism hypotheses of score-based methods do not always hold, e.g., the RNA molecule often folds into locally stable structural domains. Furthermore, almost all score-based methods use virtually the same DP algorithm to find the best-scoring structures. However, the running time of the DP algorithm is usually *O*(*n*^3^) (where *n* is the RNA sequence length), neglecting the special base pairs and weak interactions. Hence, the computational cost is not acceptable, especially when analyzing an RNA molecule longer than 1,000 nucleotides. Moreover, predicting the special base pairs in RNA structures is still a difficult task. Since an RNA structure with special bases pairs is not a nested structure in general, score-based methods have to employ sophisticated algorithms to capture these special base pairs at the cost of higher time complexity. However, the performance of these methods needs to be further improved.

In fact, it is extremely difficult to fully understand the RNA folding mechanism. ML methods, in contrast, are data driven and requiring no knowledge of such mechanism. These methods can learn the underlying folding patterns from large amount of training data. In the last few decades, ML methods have been used for many aspects of RNA secondary structure prediction methods to improve the prediction performance (see Section 4). However, they did not replace the mainstream score-based methods with respect to accuracy and generalization. This situation has been changing in the last 2 years because of the development of ML techniques, especially DL.

## ML-based methods

The ML-based methods for RNA secondary structure prediction can generally be divided into 3 categories (S1 Fig) according to the subprocess that ML participates in, i.e., score scheme based on ML, preprocessing and postprocessing based on ML, and prediction process based on ML. All the ML-based methods in these 3 categories trained their models in a supervised way [72]. These models learn functions that map inputs (features) to outputs by adjusting model parameters based on the known input–output pairs. Many of them employ free energy parameters, encoded RNA sequences, sequence patterns, or evolutionary information as key features, and their outputs can be classification labels (such as paired or unpaired) or continuous values (such as free energy). When a new input is fed to the trained model, the model can classify a corresponding label or predict a corresponding value [72].

### Score scheme based on ML

**Fig 2:**
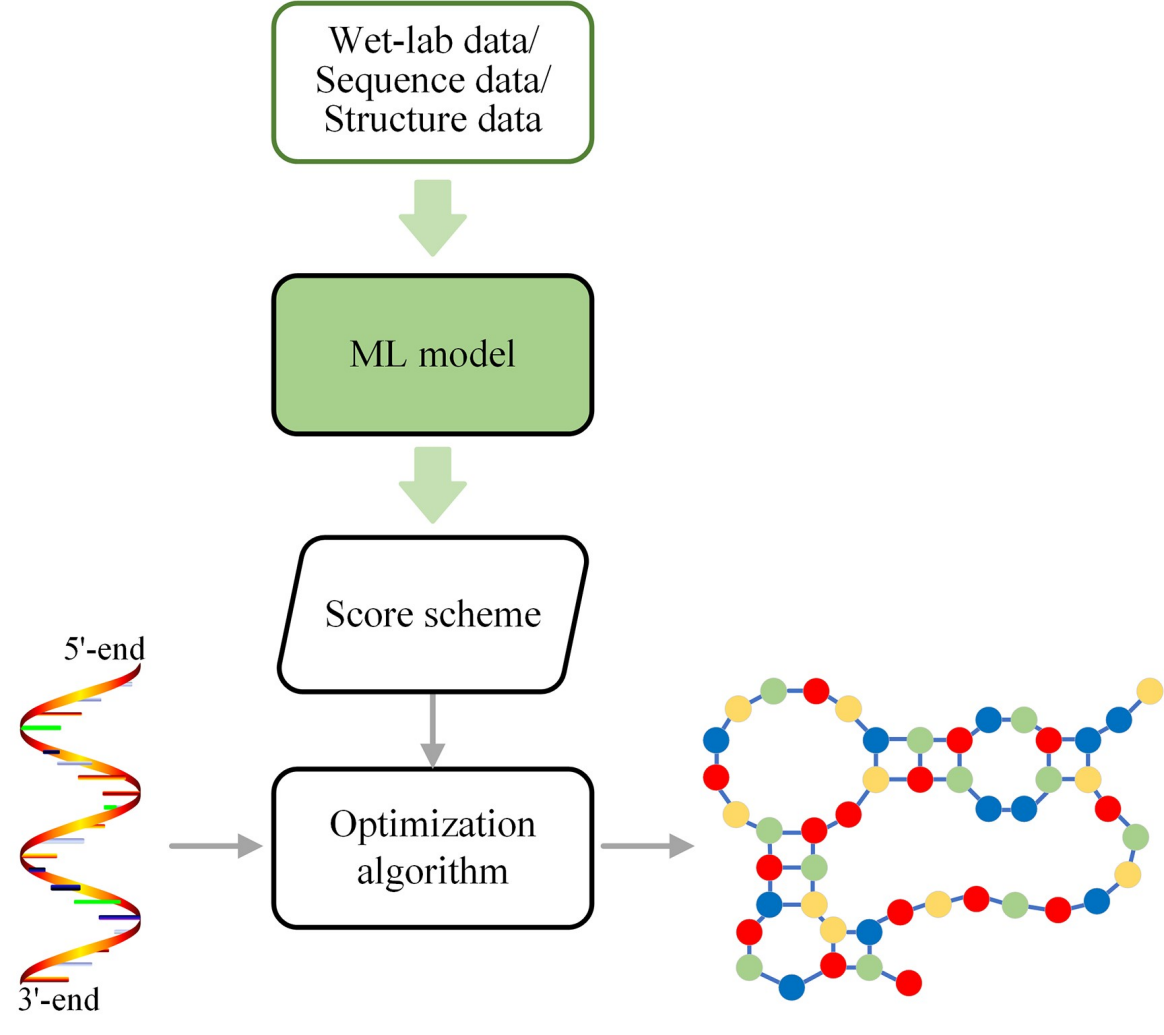
Framework for RNA secondary structure prediction methods with ML-based score schemes. Wet lab data, RNA sequence data, or RNA structure data can be employed to train an ML model to obtain a score scheme. Using this score scheme, an RNA secondary structure can be predicted using a traditional score-based approach from a single RNA sequence.

Early ML-based methods usually train an ML model that can generate a new score scheme (Fig 2) and replace the score scheme used in the traditional methods. According to the meaning of the score, ML-based score schemes can be further divided into 3 categories (S1 Fig), i.e., the free energy parameter-refining approach, weighted approach, and probabilistic approach. Although ML-based methods are used for parameter estimation in the score schemes to improve the prediction accuracy, the structure prediction is still formulated as an optimization problem, where the estimated parameters are used for the evaluation of the scores of possible conformations.

#### Free energy parameter refining based on ML

Considering the score schemes, the free energy–focused approach is the most popular approach. Ever since Tinoco and colleagues [58] put forward their hypothesis for free energy calculation (that the free energy of a secondary structure is the sum of the free energy values of its elements), many studies have been devoting their efforts to the problem of assigning free energy values to the elements of RNA molecules. Turner’s NN model [57] is the most popular approach and provides a considerably accurate approximation of the RNA free energy. However, the multiple thermodynamic parameters of the NN model have to be based on a large number of optimal melting experiments. These experiments are time and labor consuming [17,19], however, and not all free energy changes in structural elements can be measured because of the associated technical difficulties.

Some ML techniques were adopted to refine the parameters in the energy model. These techniques can employ subtle models to estimate the scores for a richer and more accurate feature representation using known thermodynamic data or RNA secondary structure data. Xia and colleagues [56] first trained a linear regression model using known thermodynamic data to infer some of the thermodynamic parameters and expanded the NN model into a more accurate model, i.e., the INN-HB model. This model provides an improved fit for the known experimental data. A disadvantage of this approach, however, is that the parameters for some structural elements are fixed before other parameters are calculated, which limits the range of possibilities considered for the overall parameter set. To overcome this problem, Andronescu and colleagues [73] proposed a constraint generation approach to estimate free energy parameters. This method uses different types of constraints to ensure that the energies of reference structures are low relative to the alternatives for the same sequence. Trained on large sets of structural and thermodynamic data, this method achieves 7% higher F-measure than the standard Turner parameters. Subsequently, the authors further modified the method and proposed a loss-augmented max-margin constraint generation model and Boltzmann-likelihood model using a larger dataset [74]. The constraints imposed on parameters ensure that the more inaccurate the structure, the greater the margin between its free energy and that of the reference structure in the training set.

Of note, the parameters determined by the above free energy parameter-refining approaches are thermodynamic and can be used directly in the algorithms embedded by the same energy model, such as miRNA target prediction [75] and RNA folding kinetics simulation [76].

#### Weighted approaches based on ML

While ML-based free energy parameter approaches successfully improved the accuracy of the RNA secondary structure prediction, the energy model is still far from ideal. Actually, the above methods for the estimation of ML-based parameters can only substitute for some wet lab experiments geared toward obtaining the energy parameters. However, it is entirely possible to obtain an improved score scheme independent of free energy based on ML techniques. Several weighted approaches were proposed that consider the parameters of RNA structure elements as weights instead of free energy changes. By removing the thermodynamic meaning, the weighted approach can utilize ML models to determine thousands of weights for more comprehensive RNA structure elements instead of obtaining a few energy parameters from a large number of wet lab experiments.

By combining a discriminative structured-prediction learning framework with an online learning algorithm, Zakov and colleagues [77] greatly increased the number of weights to approximately 70,000 by examining more types of structural elements with more numerous sequential contexts, using thousands of training datasets. Based on these weights, the ContextFold tool was proposed, marking a significant improvement in the prediction accuracy [77]. Akiyama and colleagues [78] integrated the thermodynamic approach with a structured support vector machine (SSVM) to obtain a large number of weights for detailed structure elements, of which *l*1 regularization was used to relieve overfitting. Then, MXfold was built by combining ML-based weights with experimentally determined thermodynamic parameters, achieving better performance than a model based on thermodynamic parameters or ML-based weights alone. Most recently, MXfold2 [79] was proposed by Sato and colleagues They trained a fairly deep neural network using the max-margin framework with thermodynamic regularization, which made the folding scores predicted by MXfold2 and the free energy calculated by the thermodynamic parameters were as close as possible. This method showed a robust prediction on both sequence-wise and family-wise cross-validation. These studies suggest that ML-based weights can complement the gaps in the thermodynamic parameter approach.

An advantage of the weighted approach is that it decouples structure prediction from energy estimation, which is potentially beneficial for both tasks. However, learned weights are not explainable, partly because of the black-box attribute of ML algorithms. Hence, the obtained scores cannot be used to compute the partition function, base pair binding probabilities, or centroid structures, etc.

#### Probabilistic approaches based on ML

Stochastic context-free grammars (SCFGs) are an important alternative for predicting RNA structures [29,80–84]. SCFGs specify formal grammar rules and induce a joint probability distribution over possible RNA structures for a given sequence. In particular, an SCFG model specifies a probability parameter for each production rule in the grammar and thus assigns a probability to each sequence it derives. The probability parameters are learned from datasets of RNA sequences annotated using known secondary structures, without the need for external laboratory experiments [83].

Sakakibara and colleagues [29] first applied SCFGs to tRNA secondary structure prediction. The probability parameters in their SCFG model were learned using an expectation–maximization (EM) method. Knudsen and Hein [82] improved the SCFG model by combining the evolutionary information, and, subsequently, the robust and practical tool Pfold [81] was developed. Sato and colleagues [85] proposed a nonparametric Bayesian extension of SCFGs with the hierarchical Dirichlet process to find an optimal RNA grammar from the training dataset. Using another ML model, the conditional log-linear model (CLLM), Do and colleagues [86] identified probability parameters that are most likely to discriminate correct structures from incorrect ones. CLLM is a flexible probabilistic ML model that generalizes upon SCFGs; the parameters are easily estimated, and arbitrary features can be incorporated in the model. CONTRAfold has achieved the highest single-sequence prediction accuracy to date, compared with the currently available probabilistic models. However, CLLM is very slow, which prevents its application to large training sets, and the estimated parameters have no intrinsic biological meaning. Finally, to take full advantage of the substantial numbers of RNA sequences with unknown structures, Yonemoto and colleagues [87] proposed a semi-supervised learning algorithm to obtain probability parameters in a probabilistic model that combines SCFG and a conditional random field.

However, the probabilistic approach cannot replace MFE methods for secondary structure prediction, as the accuracy of the currently best SCFG has yet to match those of the best free energy–based models. In addition, SCFG cannot describe all RNA structures, e.g., a structure containing special base pairs.

### Preprocessing and postprocessing based on ML

**Fig 3:**
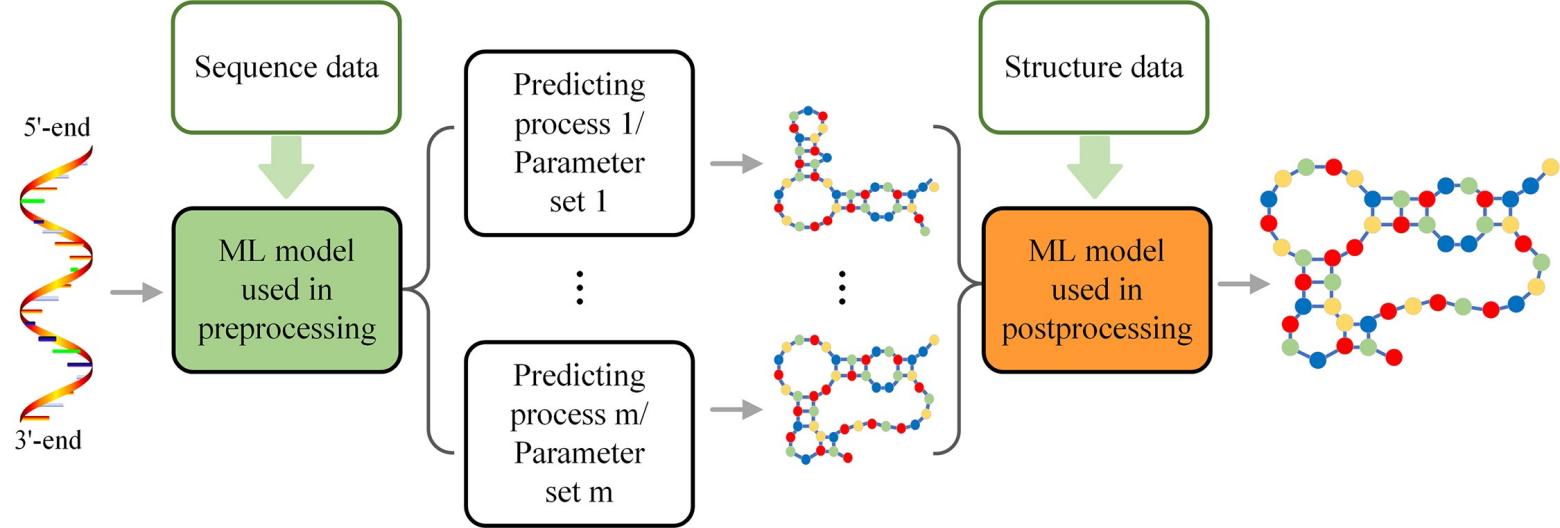
Framework for RNA secondary structure prediction methods with ML-based preprocessing or postprocessing. In RNA secondary structure prediction, ML models (trained by sequence data, in green) can be also used in pretreatment for selecting an appropriate prediction method or a group of appropriate parameters; ML models (trained by structure data, in brown) also can provide a means of determining the most likely structures among the outcomes.

ML can be also used in pretreatment, for selecting an appropriate prediction method or a group of appropriate parameters (Figs 3 and S1). A tool based on a support vector machine (SVM) was proposed by Hor and colleagues [88] for selecting the prediction method, based on the notion that different RNA sequences have different features and different prediction methods work best with specific RNA species. In another study, Zhu and colleagues [89] assumed that different RNA sequences follow different folding rules. The authors consequently proposed an SCFG model to identify the most probable folding rules before RNA secondary structure prediction.

Since different prediction methods return several different structures, the ML model can provide a means of determining the most likely structures among the outcomes (Figs 3 and S1). Combined with the graph theory, Haynes and colleagues [90] used trees to represent RNA graphical structures (edges as helices, and verticals as loops or bulges). They then trained a multilayer perceptron (MLP) model to distinguish whether a structure is RNA-like or not, using graphical invariants as input features. Assuming that a larger secondary structure is formed upon bonding of 2 smaller secondary RNA structures, Koessler and colleagues [91] also used an MLP model to predict the RNA-like probability of a structure using a special feature vector extracted from the merged trees.

### Predicting process based on ML

**Fig 4:**
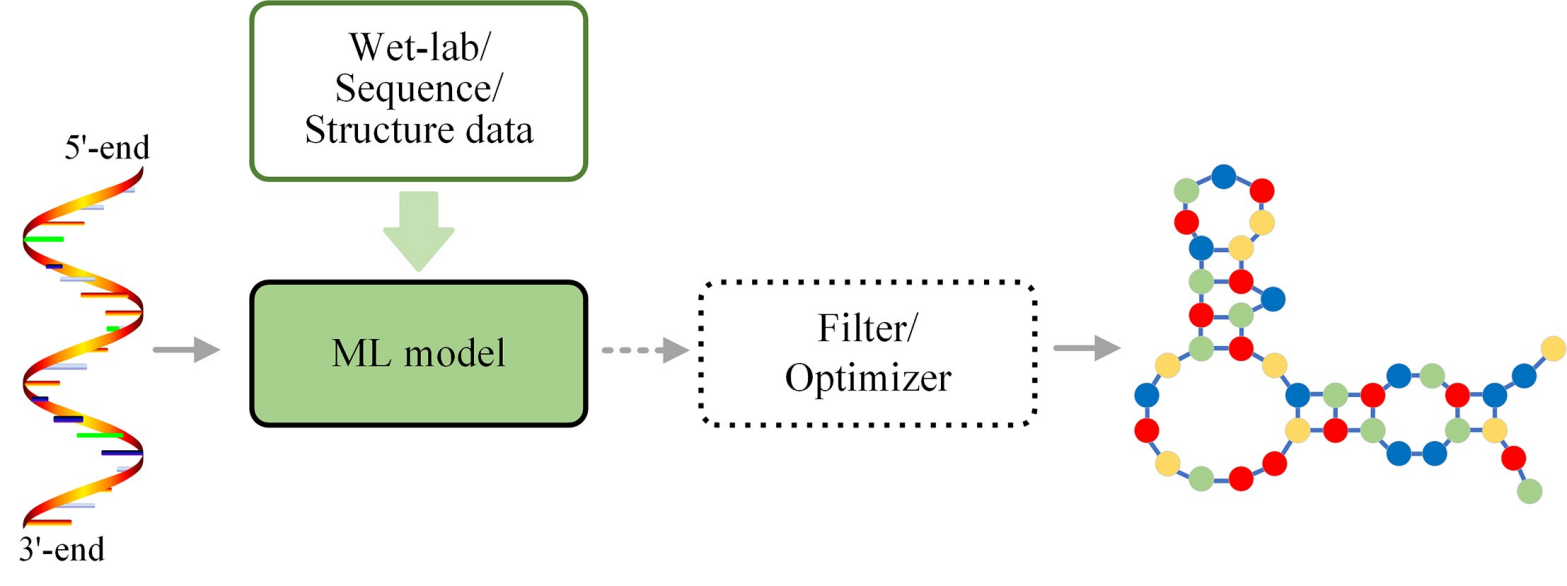
Framework for the RNA secondary structure prediction methods with ML-based prediction process. ML models (trained by wet lab, RNA sequence, or RNA structure data) are directly used to predict RNA secondary structures in an end-to-end way or followed by a filter or optimizer to obtain the optimal RNA secondary structure.

ML techniques are also directly used to predict RNA secondary structure in an end-to-end fashion or combined with other algorithms as constraints, base state detector, or structure selector. The general framework is shown in Figs 4 and S1.

#### End-to-end approach

To the best of our knowledge, the ML technique was first introduced into the RNA secondary structure predicting process by Takefuji and colleagues [92]. The authors built on Nussinov and Jacobson’s hypothesis (see Section 3.2) [52] and attempted to obtain a near-maximum independent set (MIS) from an adjacent graph (where the vertices represent base pairs, and the edges connect the incompatible vertices) using a system composed of *m* interactional neurons (*m* is the number of edges). Liu and colleagues [93] enhanced Takefuji’s work by considering the energy contribution of possible base pairs, and a Hopfield neural network (HNN) was employed to obtain MIS. However, HNN is easily trapped in local minima, limiting the accuracy of this method. To avoid this problem, Steeg and Evan [94] made use of the mean field theory (MFT) networks to identify the optimal structure, which was coupled with a sophisticated objective function with additional biological constraints. The inputs into the MFT networks are the 4 types of bases in an RNA sequence encoded in a one-hot fashion, and the output is in a format similar to CT table. Subsequently, Apolloni and colleagues [95] further developed Steeg’s method, especially with respect to the computation speed, so that it could be applied to slightly longer RNA sequences. In addition, this model uses mean field approximation to update the node in both the learning phase and the instant resolution phase. In another study, Qasim and colleagues [96] modified Takefuji’s work by building a novel MLP model to obtain MIS. This model contains *h* neurons in the hidden layer, whose activation function is based on the Kolgomorov’s theorem (*h* is the number of possible base pairs in an RNA sequence).

However, because of the relatively poor performance of the above ML models and a small amount of the available data, ML-based RNA secondary structure prediction models can only process tRNAs, with relatively low accuracy. Currently, the use of DL techniques is rising rapidly, and they are dramatically changing these circumstances. Singh and colleagues [97] proposed the first end-to-end DL model, SPOT-RNA, to predict RNA secondary structure. SPOT-RNA treats the RNA secondary structure as a CT table and employs an ensemble of ultradeep hybrid networks of ResNets and 2D-BLSTMs for the prediction. Of these, the former captures the contextual information from the whole sequence, and the latter is effective for the propagation of long-range sequence dependencies in RNA structure. Transfer learning is used to train SPOT-RNA to effectively utilize limited sample numbers. SPOT-RNA showed superior performance with several RNA benchmark datasets, greatly outperforming the best score-based methods and SCFG-based methods. Recently, the SPOT-RNA2 model [98] was proposed by the same research group. This model employed evolution-derived sequence profiles and mutational coupling as inputs and outperformed SPOT-RNA for all types of base pairs using the same transfer learning approach. E2Efold is another DL model for RNA secondary structure prediction, proposed by Chen and colleagues [99]. It integrates 2 coupled parts, i.e., a transformer-based deep model that encodes sequence information, and a multilayer network based on an unrolled algorithm that gradually enforces the constraints and restricts the output space.

In addition to the encoded RNA sequences being used as the input, other information can also be incorporated into the DL model. Calonaci and colleagues [100] trained an ensemble model based on a combination of SHAPE data, co-evolutionary data (DCA), and RNA sequence data. Their model consists of a convolutional neural network (CNN) subnetwork and an MLP subnetwork to predict penalties based on SHAPE and DCA data, respectively, with an RNAfold [17] module to generate structures using RNA sequences and penalties.

#### Hybrid approach

Alternatively, ML can be combined with other methods for a hybrid approach for RNA secondary structure prediction. Consequently, the ML model is usually considered as a scoring machine, mapping a score to each (pair of) base(s) in an RNA sequence, whose output is then passed to an independent filter to identify a reasonable structure.

Bindewald and Shapiro [49] combined an ML model and a filter to predict the consensus structure for a group of aligned RNAs. The authors chose a hierarchical network of k-nearest neighbor model to predict the possibility score for each pair of alignment columns and defined the filter by a set of rules derived from native RNA structures. Considering structure prediction as a sequence-labeling question, Lu and colleagues [101] and Wu and colleagues [102] employed a more powerful DL model, Bi-LSTM, to predict the state of each base in an RNA sequence, using a similar rule-based filter to deal with conflicting pairing. Differently from the above studies, Bi-LSTM was used as a structure filter in DpacoRNA [103], and a parallel ant colony optimization method was used to predict the most probable structures. Another type of an ML-based hybrid approach combines ML models and optimization methods. Liu’s group [104] used a CNN model to predict the status distribution of each base in an RNA sequence, and a DP algorithm was employed to find the maximum probability structure. The same group [105] also used the Bi-LSTM model instead and another optimization algorithm, similar to that used in [106]. Instead of developing a new optimizer, Willmott and colleagues [107] utilized an existing SHAPE-directed method (SDM) [108] as the optimizer, which can predict optimal structure from SHAPE data, and trained a Bi-LSTM model to generate SHAPE-like data (i.e., determine the state of each nucleotide) of an RNA sequence as the inputs of SDM.

Compared with the end-to-end approach, the performance of the hybrid approach is relatively poor, perhaps because of a bias between the training objective of the ML part and the overall system objective. Most methods in the hybrid approach are trained and tested using small-scale datasets. Hence, generalization of their abilities requires further verification.

## Discussions

It is well known that transcript abundance helps to identify transcripts of interest under different conditions, while the RNA structure helps to explain how these transcripts function. An excellent RNA structure prediction method is not only important for inferring RNA function, but also relates to many downstream studie*s*, including ncRNA detection [109–111], folding dynamics simulations [112], hybridization stability assessment [113], and oligonucleotide [114,115] or drug design [116–120]. It is worth noting that RNA secondary structure prediction is also a useful tool for studying viruses, such as the SARS-CoV-2 virus responsible for the current pandemic [121,122].

### The advantages of ML-based methods

Compared with comparative sequence analysis and traditional score-based methods, ML-based methods have some advantages. First, ML-based methods do not necessarily rely on the biological mechanism, which is usually difficult to thoroughly understand. Instead, they can utilize the information contained in various types of data, and, therefore, performance limitation caused by the mechanism hypothesis can be circumvented. ML-based methods can also be easily coupled with known biological mechanisms. Further, in terms of prediction performance, where a large amount of data is available, models with no or little knowledge of biological mechanisms usually perform better than mechanism-dependent ones. This also suggests that the assumed mechanism of RNA folding may be incomplete or not accurate. Second, in contrast to traditional score-based methods, the end-to-end DL methods do not need to consider the difficulties caused by base matching rules. Traditional score-based methods employ sophisticated algorithms to satisfy base matching rules at the cost of high time complexity. However, without the constraint of these rules, end-to-end models [97] can train and predict all the base pairs in RNA structures, regardless of whether the base pairs associate with secondary or tertiary interactions. Third, compared with traditional methods, the ML-based methods can be considerably flexible. The inputs of ML-based models can be either one-dimensional or multidimensional, extracted features or encoded bases, and homogeneous data or heterogeneous data, and the outputs can be CT tables, labeled sequences, nucleotide states, or free energy values. In addition, the construction of the ML models is diverse, from simple Hopfield networks to complex ensemble deep neural networks. Fourth, once the model training is completed, the ML-based end-to-end prediction methods run very fast. Unlike DP algorithm, the time complexity of ML models is independent of the input scale, which is advantageous when dealing with long RNAs.

### Datasets and their impacts on ML-based methods

Today, many public RNA structure databases and other related datasets are available online, which provide abundant data for model training. Generally, these databases can be classified into 2 types, i.e., comprehensive databases and dedicated databases. A comprehensive database often consists of RNA structures with different conformations and in different RNA species, for example, RNA Strand (4,666 RNAs available) [123], RCSB Protein Data Bank (PDB, 4,962 RNAs available) [124], and bpRNA-1m (102,348 RNAs available) [125]. Some of these databases (e.g., PDB) collect tertiary structures obtained by wet lab experiments, while others obtained data using comparative sequence analysis method (less accurate than those obtained by wet lab experiments, e.g., pbRNA-1m). Dedicated databases generally involve only a single RNA species (tRNA [126], rRNA [127], or tmRNA databases [128]) or a single type of RNA structure (such as loop [129], pseudoknot [130], or noncanonical base pair [131]) generally. Based on these dedicated databases, some public benchmark datasets were established, such as ArchiveII [132] and RNAStralign [133]. These datasets are generally composed of tens of thousands of RNAs in different RNA species (rRNA, tRNA, SRP, tmRNA, etc.). In addition, other databases used in ML-based methods are Rfam [51] and NNDB [57], which provide RNA family information and thermodynamic parameters, respectively.

Data are extremely important for building ML-based RNA secondary prediction models, especially DL-based models with a large number of parameters. One of the reasons that the recent DL-based methods [79,97,99] outperform the traditional ML-based models is the improvement of the quality and quantity of the training sets. It is worth noting that the performance of DL-based methods may be overestimated due to the data similarity between the training and test set. Most of studies only ensured that the RNAs in test sets of these methods were not so similar (80% similarity [134] as a cutoff typically) to those in the training sets, but RNAs from the same families were not explicitly excluded from the testing set. The sequences and structures in the same RNA family are similar, resulting that the model performance obtained on testing sets is better than reality [79,97].

Another issue that may affect the model performance is the imbalanced RNA families in training sets, e.g., thousands of 16S rRNAs but only a small number of telomerases occur in one dataset. When the length of the input RNA is comparable, trained models tend to perform better on the RNA species that are more prevalence in the training set [99]. How to deal with unbalanced data is an active topic in the ML community. Study [99] adopted an up-sampling strategy to balance the RNAs in different families, and their model performance was further improved.

Generally, the enhancement of predictive ability is associated with the relatively large scale of the ML model, which requires large amounts of data for parameter training. Although a large number of RNA structure data in various formats is available, these are insufficient in terms of training large-scale DL models, especially with respect to the availability of high-accuracy data. Hence, questions on how to effectively utilize the limited data and cope with overfitting of a large-scale DL model are also important issues that remain to be resolved.

### Current pending challenges

Enormous progress has been made toward predicting RNA secondary structure by using ML-based methods. These methods are state of the art when considering most indices of prediction performance. However, some issues still require resolving.

First, the accuracy of prediction should be improved further. Sato and colleagues [79] used the RNAs in the newly discovered RNA families to form an independent test set (not used in all the tested methods), and based on this dataset, a rigorous test was performed among 6 most accurate RNA secondary structure prediction methods. The test results showed that, among these methods, the highest positive predictive value (PPV) is 0.636 (achieved by TORNADO) [84], the highest sensitivity is 0.720 (achieved by RNAfold) [17], and the highest F value is 0.632 (achieved by MXfold2) [79]. Using another independent dataset collected from PDB, Singh and colleagues [98] performed a comprehensive comparison among 27 kinds of well-known RNA secondary structure prediction methods. Their results showed when homologous sequences were available, the highest F value and sensitivity achieved were 0.774 and 0.727, respectively (both by SPOT-RNA2). These results objectively show that there is still much room for improvement in RNA secondary structure prediction. Moreover, many traditional methods neglect special base pairs to avoid a large number of false positives or to limit computational complexity [71,135]. While some methods can predict RNA secondary structures containing pseudoknots [46] or noncanonical base pairs [63], none of them can predict both. Although the recently proposed ML-based methods can predict all kinds of special base pairs, the special base pair prediction accuracy is still limited.

The RNA sequence length limitation is another intractable issue, which becomes quite problematic with the recently discovered long (1,000 to 10,000 nt) ncRNA [136]. Although ML-based methods do not suffer from high time complexity as most score-based methods do, they are unable to effectively capture such long-range interactions within an RNA sequence. On the other hand, training an ML model with such a large-scale input consumes a huge amount of computational resources and is often unrealistic.

For ML-based RNA secondary structure prediction models, overfitting is a very important issue [84], especially for DL-based models with a large number of parameters. The overfitted models perform well on the test RNAs similar to that in the training data but generalize poorly on dissimilar ones. It seems that they only memorize the secondary structure of RNAs in the training data, rather than actually learn the folding mechanism from them. A result in paper [79] showed that E2Efold [99] outperformed many traditional methods on the dataset ArchiveII but performed poorly on the RNAs from newly discovered RNA families. This suggested that E2Efold might suffer from a heavy overfitting. Similarly, another paper [137] reported that the F score of ContextFold also lowered by 24% when testing on a set of structurally dissimilar RNAs to the training set. Although most DL-based methods take many precautions to alleviate overfitting by many techniques (such as using regularization [100], enlarging dataset [97], adding constraints [99], or combining Turner’s nearest neighbor free energy parameters), the concerns about overfitting remain.

At last, the folding mechanisms need further exploration. Traditional RNA secondary structure prediction is based on different RNA folding mechanism hypotheses (S1 Table), while data-driven ML-based methods can learn such mechanism implicitly from known data based on different RNA sequences or sequence features. However, to the best of our knowledge, few folding mechanisms have been revealed from the established ML-based models, although great advances have been made in terms of prediction accuracy. Part of the reason is that the interpretability [138] of DL models is still a challenge today.

### Future trends of development

Currently, RNA secondary structure prediction is successfully shifting toward ML-based approaches, away from traditional score-based methods, and DL will surely continue to improve the prediction performance. The subtle structure of the DL model is a prerequisite to this end. Since the DL model is being rapidly developed in the natural language processing and image processing fields, using mature DL blocks from these fields, or combining them in such fields constitutes a feasible way to generate an excellent DL model for RNA secondary structure prediction.

Further, using a DL model to predict the free energy parameter is an inevitable trend for more accurate energy estimations, when additional wet lab experimental data become available. However, these parameters may not improve RNA secondary prediction accuracy because they have to be combined with traditional score-based methods. On the other hand, combing an ML-based method and an optimization method is a promising approach for improving prediction performance.

## Conclusions

RNA structure is one of the central pieces of information for understanding biological processes, and determining RNA secondary structure will continue to be a hot topic in the computation and biology fields. In this review, we focused on ML-based methods, which involve many aspects of RNA secondary structure prediction. ML techniques have greatly improved the performance of prediction methods, including accuracy, applicability, and running speed. However, to thoroughly resolve the RNA secondary structure prediction problem, a more subtle ML model is still needed. At the moment, ML-based methods cannot be used as substitutes for wet lab experiments for obtaining high-resolution structures. Nonetheless, the advent of DL technologies and high-performance hardware will foster a new generation of RNA secondary prediction tools with an improved accuracy and running speed.

## Supporting information

S1 Fig Classification of ML-based RNA secondary structure prediction methods. According to the subprocess that ML participates in, the ML-based RNA secondary structure prediction methods were classified into 3 categories, i.e., score scheme based on ML (containing 3 subcategories: free energy–refining approach, weighted approach, and probabilistic approach), preprocessing and postprocessing based on ML (containing 2 subcategories: preprocessing and postprocessing), and prediction process based on ML (containing 2 subcategories: end-to-end approach and hybrid approach). (TIF)

S1 Table Comparison of RNA secondary structure prediction methods. (DOCX)
