# Supplementary figures and images for: Review of machine learning methods for RNA secondary structure prediction
Source: PLoS Comput Biol. 2021 Aug 26;17(8):e1009291. doi: 10.1371/journal.pcbi.1009291 (PMC8389396; doi:10.1371/journal.pcbi.1009291)

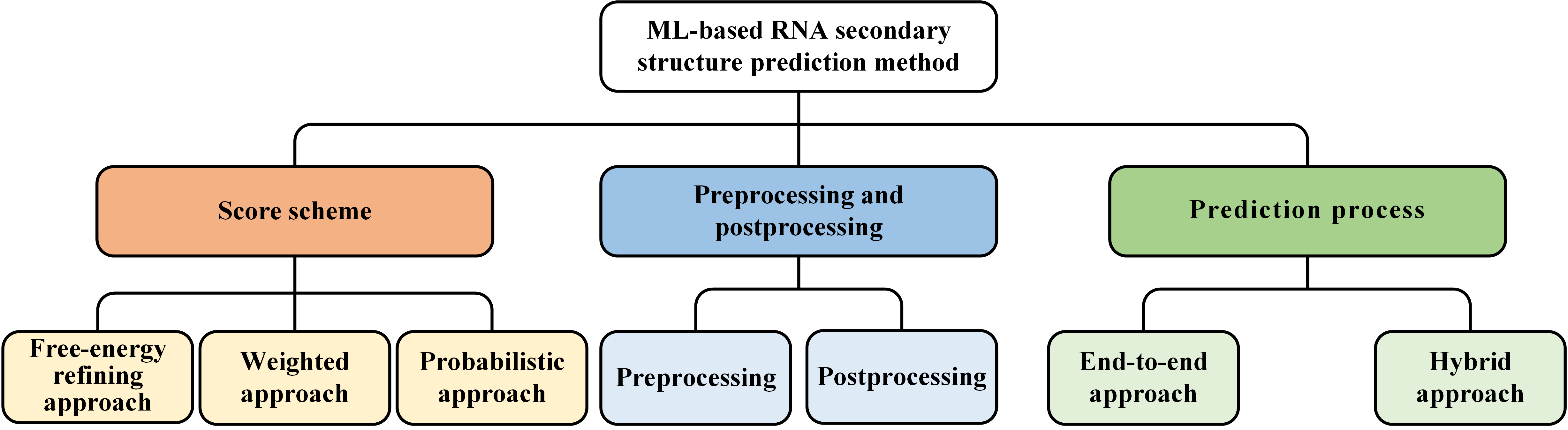

Supplement: S1 Fig — (TIF) [file pcbi.1009291.s001.tif]
