# Supplementary material for: Review of machine learning methods for RNA secondary structure prediction
Source: PLoS Comput Biol. 2021 Aug 26;17(8):e1009291. doi: 10.1371/journal.pcbi.1009291 (PMC8389396; doi:10.1371/journal.pcbi.1009291)
Supplement: S1 Table — (DOCX) [file pcbi.1009291.s002.docx]

**Supplemental Table 1. Comparison of RNA secondary structure prediction methods**

| **Method** | **Category** | **Hypothesis** | **Algorithm** | **Algorithm mechanism** |
| --- | --- | --- | --- | --- |
| Traditional computational methods | Comparative sequence analysis | RNA secondary structure is evolutionarily conserved | Optimization Algorithm (*e.g.*, divide and conquer) | Find base pairs that covary to maintain base complementarities of a given sequence, using a set of homologous sequences |
|  | Score-based method | RNA structure is a structure with a minimum/maximum total score (*e.g.*, free energy minimization) | Optimization Algorithm (*e.g.*, dynamic programming) | Find the structure with the optimal score recursively |
| ML-based methods | Score scheme based on ML model | Depending on the other part of the algorithm (*e.g.*, total score minimization) | Regression Model (*e.g.*, linear regression) | Train a regression model to learn the relation between an input motif and the output score |
|  | Preprocessing and postprocessing based on ML model | Depending on the other part of the algorithm (*e.g.*, free energy minimization) | Classification model (*e.g.*, SVM) | Train a classification model to learn the relation between an input sequence and the corresponding parameter group, or classify an input structure as a native or nonnative structure |
|  | Predicting process based on ML model | No hypothesis | Classification model (*e.g.*, LSTM) | Train a classification model to learn the relation between input sequence and output structure |
